# Effectiveness of the 23-valent pneumococcal polysaccharide vaccine against vaccine serotype pneumococcal pneumonia in adults: A case-control test-negative design study

**DOI:** 10.1371/journal.pmed.1003326

**Published:** 2020-10-23

**Authors:** Hannah Lawrence, Harry Pick, Vadsala Baskaran, Priya Daniel, Chamira Rodrigo, Deborah Ashton, Rochelle C. Edwards-Pritchard, Carmen Sheppard, Seyi D. Eletu, David Litt, Norman K. Fry, Samuel Rose, Caroline Trotter, Tricia M. McKeever, Wei Shen Lim

**Affiliations:** 1 Department of Respiratory Medicine, Nottingham University Hospitals NHS Trust, Nottingham, United Kingdom; 2 Division of Epidemiology and Public Health, University of Nottingham, Nottingham, United Kingdom; 3 NIHR Nottingham Biomedical Research Centre, Queen’s Medical Centre, Nottingham, United Kingdom; 4 Department of Respiratory Medicine, University Hospitals of Derby and Burton NHS Foundation Trust, Derby, United Kingdom; 5 Division of Respiratory Medicine, University of Nottingham, Nottingham, United Kingdom; 6 Respiratory and Vaccine Preventable Bacteria Reference Unit, Public Health England–National Infection Service, Colindale, London, United Kingdom; 7 Immunisation and Countermeasures Division, Public Health England Colindale–National Infection Service, London, United Kingdom; 8 Disease Dynamic Unit, Department of Veterinary Medicine, University of Cambridge, Cambridge, United Kingdom; Universitair Medisch Centrum Utrecht, NETHERLANDS

## Abstract

**Background:**

Vaccination with the 23-valent pneumococcal polysaccharide vaccine (PPV23) is available in the United Kingdom to adults aged 65 years or older and those in defined clinical risk groups. We evaluated the vaccine effectiveness (VE) of PPV23 against vaccine-type pneumococcal pneumonia in a cohort of adults hospitalised with community-acquired pneumonia (CAP).

**Methods and findings:**

Using a case-control test-negative design, a secondary analysis of data was conducted from a prospective cohort study of adults (aged ≥16 years) with CAP hospitalised at 2 university teaching hospitals in Nottingham, England, from September 2013 to August 2018. The exposure of interest was PPV23 vaccination at any time point prior to the index admission. A case was defined as PPV23 serotype-specific pneumococcal pneumonia and a control as non-PPV23 serotype pneumococcal pneumonia or nonpneumococcal pneumonia. Pneumococcal serotypes were identified from urine samples using a multiplex immunoassay or from positive blood cultures. Multivariable logistic regression was used to derive adjusted odds of case status between vaccinated and unvaccinated individuals; VE estimates were calculated as (1 − odds ratio) × 100%. Of 2,357 patients, there were 717 PPV23 cases (48% vaccinated) and 1,640 controls (54.5% vaccinated). The adjusted VE (aVE) estimate against PPV23 serotype disease was 24% (95% CI 5%–40%, *p =* 0.02). Estimates were similar in analyses restricted to vaccine-eligible patients (*n =* 1,768, aVE 23%, 95% CI 1%–40%) and patients aged ≥65 years (*n =* 1,407, aVE 20%, 95% CI −5% to 40%), but not in patients aged ≥75 years (*n =* 905, aVE 5%, 95% CI −37% to 35%). The aVE estimate in relation to PPV23/non-13-valent pneumococcal conjugate vaccine (PCV13) serotype pneumonia (*n* = 417 cases, 43.7% vaccinated) was 29% (95% CI 6%–46%). Key limitations of this study are that, due to high vaccination rates, there was a lack of power to reject the null hypothesis of no vaccine effect, and that the study was not large enough to allow robust subgroup analysis in the older age groups.

**Conclusions:**

In the setting of an established national childhood PCV13 vaccination programme, PPV23 vaccination of clinical at-risk patient groups and adults aged ≥65 years provided moderate long-term protection against hospitalisation with PPV23 serotype pneumonia. These findings suggest that PPV23 vaccination may continue to have an important role in adult pneumococcal vaccine policy, including the possibility of revaccination of older adults.

## Abstract 2

Hannah Lawrence and colleagues assess the long-term vaccine effectiveness of PPV23 in adults in the setting of an established childhood pneumococcal vaccine program.

## Author summary

**Why was this study done?:**

- *Streptococcus pneumoniae* is the commonest bacterial cause of community-acquired pneumonia (CAP) worldwide with over 90 different serotypes.
- A 23-valent pneumococcal polysaccharide vaccine (PPV23) targeting 23 common serotypes is recommended for use in adults in various countries to protect against pneumococcal infection.
- The long-term vaccine effectiveness (VE) of PPV23 against vaccine serotype pneumococcal CAP in adults in the setting of an established childhood pneumococcal vaccine programme is not known.

**What did the researchers do and find?:**

- We retrospectively analysed data from a cohort of adults hospitalised with CAP in Nottingham, England, who had a diagnostic blood or urine test to determine (i) whether they had pneumococcal disease and (ii) if so, whether or not it was a serotype covered by the PPV23 vaccine.
- We calculated the VE of PPV23 in our cohort by calculating the odds of infection with vaccine-type pneumococcal pneumonia (cases) versus pneumonia of an alternate cause (controls) between vaccinated and unvaccinated individuals.
- In our group of 2,357 patients (717 PPV23 cases, 1,640 controls) with an average time of 10 years since PPV23 vaccination, we estimated the VE of PPV23 against PPV23 serotype pneumonia to be 24% after adjustment for patient factors (95% CI 5%–40%, *p* = 0.02).

**What do these findings mean?:**

- PPV23 vaccination provides moderate long-term protection against hospitalisation with PPV23 serotype pneumonia.
- PPV23 vaccination may continue to have an important role in national pneumococcal immunisation policies, including the possibility of revaccination of older adults.

## Introduction

*Streptococcus pneumoniae* is widely accepted as the most common bacterial cause of community-acquired pneumonia (CAP) worldwide and is associated with substantial morbidity, mortality, and economic burden [1,2]. Two different types of pneumococcal vaccine are currently available: the 23-valent pneumococcal polysaccharide vaccine (PPV23) and pneumococcal conjugate vaccines (PCVs). In the UK, a national pneumococcal vaccination policy with 7-valent PCV was introduced for children under 2 years old in September 2006 and replaced with the 13-valent PCV in 2010 [3]. Subsequent reductions in invasive pneumococcal disease (IPD) and nasopharyngeal carriage due to vaccine serotypes in children were observed [4]. Reductions in vaccine type IPD and non-invasive pneumococcal pneumonia (NIPP) in adults followed, largely due to herd protection effects [4]. However, with the emergence of replacement serotypes in the UK, recent studies have observed increases in the incidence rates of IPD and pneumococcal pneumonia due to non-PCV13 serotypes [5,6].

Vaccination with PPV23, containing the PCV13 serotypes (except 6A) and 11 additional serotypes (2, 8, 9N, 10A, 11A, 12F, 15B, 17F, 20, 22F, and 33F) has been available in England to those ≥65 years and those in a clinical risk group since 2003, with coverage in those ≥65 years at 69.5% in March 2018 [7]. PPV23 vaccination has been found to be effective in preventing IPD and displays a waning effect with time from vaccination [8,9]. However, the effectiveness of PPV23 against pneumococcal pneumonia is controversial [10]. There are scant data regarding PPV23 serotype-specific vaccine effectiveness (VE) against NIPP in the setting of a well-established national infant pneumococcal vaccination programme. Such data are important to inform future adult vaccination policies [11].

The aim of this work was to evaluate the VE of PPV23 against vaccine-type pneumococcal pneumonia in adults hospitalised with CAP. Secondary aims were to (i) estimate VE in defined patient subgroups, (ii) estimate VE against pneumococcal serotypes not covered by herd protection from PCV13 (PPV23/non-PCV13 pneumonia), and (iii) examine the effect of time since vaccination on VE.

## Methods

### Study design

This study is a secondary analysis of data collected from a prospective observational cohort study of consecutive adult patients with CAP admitted to 2 large university hospitals in Nottingham, UK, between September 2013 and August 2018. The primary study was designed to determine trends in pneumococcal serotypes in adults hospitalised with CAP over time; study details including epidemiological results arising over the first 10 years of study have been published previously [6,12]. Ethical approval for the primary study was provided by the Nottingham Research Ethics Committee (REC reference 08/H0403/80). For this analysis, as with previous influenza and pneumococcal vaccine studies estimating VE in a real-world population, a nested case-control test-negative design was used [13,14]. The exposure of interest was PPV23 vaccination prior to the index admission, and the primary outcome was PPV23 vaccine serotype pneumococcal pneumonia. The study is reported as per the Strengthening the Reporting of Observational Studies in Epidemiology (STROBE) guideline (S1 Text).

### Study cohort

Study eligibility criteria, recruitment, and microbiological processes have been described in full previously [6]. Briefly, patients aged ≥16 years presenting with one or more acute lower respiratory tract symptoms, evidence of acute infiltrates consistent with respiratory infection on admission chest radiograph, and treated for a diagnosis of CAP were eligible. Exclusion criteria included prior hospitalisation within 10 days of index admission, a diagnosis of tuberculosis, or a diagnosis of post-obstructive pneumonia. Following informed consent, information on demographics and clinical characteristics (including potential confounders) were collected using a standardised proforma via researcher interview and medical records. For this analysis, only patients providing a sample subjected to pneumococcal serotype-specific testing were included. Pneumococcal serotype was identified using the following: (i) for bacteraemic cases: slide agglutination tests with latex antisera (ImmuLex Pneumotest kit, SSI Diagnostica, Hillerød, Denmark) or standard factor sera (SSI Diagnostica), or (from October 2017) whole genome sequencing; (ii) for NIPP cases: multiplex immunoassay (Bio-plex24) applied to urine samples to detect pneumococcal serotypes 1, 2, 3, 4, 5, 6A, 6B, 7F, 8, 9N, 9V, 10A, 11A, 12F, 14, 15B, 17F, 18C, 19A, 19F, 20, 22F, 23F, 33F and the pneumococcal cell-wall polysaccharide plus some cross-reactive serotypes [6,15,16].

### Case groups

The primary case group of interest was patients with pneumococcal pneumonia caused by PPV23 vaccine serotypes. The secondary case group comprised patients with PPV23/non-PCV13 serotype pneumonia (2, 8, 9N, 10A, 11A, 12F, 15B, 17F, 20, 22F, 33F); cases caused by PPV23/PCV13 serotypes were censored from analysis of the secondary group.

### Control group

A patient with non-PPV23 vaccine serotype pneumococcal disease or nonpneumococcal pneumonia was defined as a control. This included Bio-plex24 negative cases (pneumonia of alternate aetiology), Bio-plex24 assay common polysaccharide (CPS)-antigen–only positive cases, and non-PPV23 vaccine-type *S*. *pneumoniae* cases. No matching of cases with controls was conducted. The control group remained the same for both primary and secondary analyses and was restricted as appropriate for subgroup analyses.

### Multiple serotypes identified

Where multiple serotypes were identified in a single patient, these were excluded from the primary analysis if the identified serotypes crossed the case-control definition. For analysis of the PPV23/non-PCV13 group, a case was included if one of the identified serotypes fulfilled the case definition and none of the identified serotypes fulfilled the definition of a control.

### Vaccine status

At the time of hospital admission, patient self-reported pneumococcal vaccine status was recorded. Date of vaccination was confirmed from primary care records where available. In the primary analysis, patients were considered vaccinated if (i) vaccine status was confirmed via primary care records or (ii) they self-reported having had the vaccine. Details on influenza vaccination (a potential confounding variable) were also collected. A patient was considered vaccinated against influenza if they had received the influenza vaccine in the 12 months prior to index admission only (confirmed and self-reported). Sensitivity analysis of the primary outcome including only patients with vaccine status confirmed via primary care records was performed. Cases with vaccination less than 14 days prior to disease were excluded.

### Statistical analysis

The case and control groups and vaccinated and unvaccinated individuals were compared using the appropriate summary statistic for the variable (proportions for binary variables, median and interquartile range [IQR] for non-normally distributed continuous variables). Odds ratios with 95% CIs and *p*-values for significance testing were calculated for binary variables. Logistic regression and chi-squared tests for trend were used to test associations between ordered categorical exposure variables (severity category, baseline performance status as defined by the ECOG Performance Scale) [17] and binary outcomes. Patients with missing data on vaccine status were excluded from the primary analysis. To investigate reporting bias, sensitivity analysis was performed by including this group as either vaccinated or unvaccinated in turn.

Adjusted odds ratios were derived using multivariable logistic regression models to describe the odds of case status between vaccinated and unvaccinated individuals; the outcome variable was case versus control. For the main analysis following modelling using Directed Acyclic Graphs (www.dagitty.net) [18], confounders included in the model a priori were age, sex, flu vaccination status in the past year, and clinical at-risk groups defined in accordance with Public Health England’s ‘Immunisation against Infectious Diseases’ (The Green Book) [3]. Influenza vaccination was included as an a priori confounder due to evidence that it is associated with PPV23 uptake and linked with health-seeking behaviours [19]. Smoking status was tested as an adjustment variable; it did not alter the results and so was not included. To account for change in serotype distribution over study years, year of index admission was tested as an adjustment variable; it did not alter the results and was not included in the final model. Likelihood ratio testing of continuous variables was performed to determine best fit (continuous versus grouped). VE estimates were calculated as (1 − odds ratio) × 100%. Subgroup analyses were performed with the whole cohort (cases and controls) restricted to those who were: (i) vaccine eligible under current UK pneumococcal vaccine policy, (ii) those ≥65 years old, and (iii) those ≥75 years old.

A secondary analysis examining the effect of time since vaccination on VE including patients with confirmed vaccine status only was performed using a categorical variable with 5 levels for time interval between vaccination date and index admission (never vaccinated, vaccinated 0–5 years, 5–10 years, 10–15 years, and ≥15 years prior to admission). A logistic regression model was used to derive the odds of being a case in each vaccination category compared to those never vaccinated. A *p*-trend across the groups was calculated using the likelihood ratio test. To further investigate long-term decline in VE, a categorical variable for each individual year from vaccination to index admission (up to 24 years) and a cubic spline model were calculated with knots at 1, 4, and 8 years [9]. All analyses were performed using Stata 16 [20]. The study was conceived in 2017, and a prospective analysis plan was written by HL, TM, and WSL in March 2019 (S2 Text). Following peer review, a serotype-specific VE analysis and an analysis of all PPV23 cases excluding serotype 5 were performed.

## Results

### Cohort description

During the 5-year study period, of 2,447 eligible study participants, 54 were excluded as no vaccine status was available, leaving 2,393 patients. In this cohort of predominantly NIPP, pneumococcal serotype was detected by Bio-plex24 assay in 968 (40.5%) and by blood culture in 110 (4.6%) patients, respectively. In 36 patients, multiple serotypes crossing the case-control definition were detected, leaving 2,357 patients for the primary analysis. The most common serotypes detected were serotype 3 (*n =* 197), 8 (*n =* 192), 12F (*n =* 60), 15A (*n =* 54), and 5 (*n =* 41).

### Comparison of the vaccinated versus unvaccinated groups

Of 2,357 patients, vaccine status was obtained from primary care records in 1,820 (77.2%) patients and was self-reported in 537 (32.8%). Mean time between vaccination and index admission was 10.3 (SD 5.8) and 10.4 (SD 5.2) years in the cases and controls, respectively. The shortest interval between vaccination and index admission was 47 days. Vaccinated patients were older (74.1 versus 57.4 years, *p* < 0.001) with a poorer baseline performance status (*p*-trend < 0.0001) and higher severity disease on admission (29.7% versus 15.5% high severity by CURB65 category; *p*-trend < 0.001) (S1 Table). They were more likely to have comorbid diseases except liver disease, alcohol dependence, and asthma. Prior vaccination with PCV13 in our cohort was very low at <0.5%.

### Comparison between cases of PPV23 serotype pneumonia and controls

There were 717 cases of PPV23 serotype pneumonia (48% vaccinated) and 1,640 controls (54.5% vaccinated). Compared to controls, cases were of a similar age (66.5 versus 65.4 years, *p =* 0.18) but were less likely to be male (47.6% versus 56.9%, *p* < 0.0001) (Table 1). Cases had a better baseline performance status (*p*-trend = 0.01), had higher severity disease on admission (26.2% versus 21.5% high severity by CURB65; *p*-trend = 0.01), were less likely to have malignancy or cardiac disease, but were more likely to be alcohol dependent.

**Table 1:** Characteristics of cases and control groups for the primary analysis.

	Controls *N* (%)	Case PPV23 Disease *N* (%)	Odds Ratio (95% CI)	*p*-Value
***Number***	1,640	717		
***Mean Age (SD)***	66.5 (18.3)	65.4 (18.7)	1.00 (0.99–1.00)	0.18
***Sex*, *Male***	932 (56.9)	341 (47.6)	0.69 (0.58–0.82)	**<0.001**
***Residential Care***	57 (3.5)	24 (3.4)	0.96 (0.59–1.56)	0.88
***Baseline Performance Status***				
**0**	522 (31.8)	285 (39.8)	1	
**1**	587 (35.8)	229 (31.9)	0.71 (0.58–0.88)	
**2**	291 (17.7)	123 (17.1)	0.77 (0.60–1.00)	
**3**	81 (4.9)	31 (4.3)	0.70 (0.45–1.09)	
**4**	55 (3.4)	16 (2.2)	0.53 (0.30–0.95)	0.009*
**Missing**	104 (6.3)	33 (4.6)		
***Severity by CURB65 Score***				
**Low**	820 (50.0)	313 (43.7)	1	
**Moderate**	467 (28.5)	216 (30.1)	1.21 (0.98–1.49)	
**Severe**	353 (21.5)	188 (26.2)	1.40 (1.12–1.74)	0.009*
***Comorbidity***				
**Malignancy**	168 (10.3)	52 (7.3)	0.68 (0.49–0.95)	**0.02**
**Liver disease**	31 (1.9)	19 (2.7)	1.41 (0.79–2.52)	0.24
**Cardiac failure**	112 (6.8)	33 (4.6)	0.66 (0.44–0.98)	**0.04**
**Cerebrovascular disease**	127 (7.7)	52 (7.3)	0.93 (0.67–1.30)	0.68
**Renal disease**	157 (9.6)	67 (9.3)	0.97 (0.72–1.32)	0.86
**Diabetes**	266 (16.2)	110 (15.3)	0.94 (0.73–1.19)	0.59
**IHD**	188 (11.5)	63 (8.8)	0.74 (0.55–1.00)	**0.05**
**Cognitive impairment**	55 (3.4)	26 (3.6)	1.08 (0.67–1.74)	0.74
**Asthma**	163 (9.9)	84 (11.7)	1.20 (0.91–1.59)	0.19
**COPD**	393 (24.0)	169 (23.6)	0.98 (0.80–1.20)	0.84
**Chronic heart disease**	277 (16.9)	86 (12.0)	0.67 (0.52–0.87)	**0.003**
**Chronic lung disease**	446 (27.2)	192 (26.8)	0.98 (0.80–1.19)	0.83
**Hypertension**	389 (23.7)	182 (25.4)	1.09 (0.89–1.34)	0.39
**Alcohol**	34 (2.1)	26 (3.6)	1.78 (1.06–2.99)	**0.03**
**Immunosuppression**	75 (4.6)	33 (4.6)	1.01 (0.66–1.53)	0.98

*p-Trend derived from chi-squared test for trend.

Patient characteristics in the control and case groups. Unadjusted odds ratios with 95% CIs and *p*-values are presented (*p*-values in bold <0.05). The baseline group for comparison is the control group in all analysis.

**Abbreviations:** COPD, chronic obstructive pulmonary disease; IHD, ischaemic heart disease; PPV23, 23-valent pneumococcal polysaccharide vaccine

### Primary analysis: VE against PPV23 serotypes

In the primary analysis of all cases of PPV23 serotype disease, the crude VE estimate was 23% (95% CI 8%–35%) (Table 2). Following adjustment for age, sex, flu vaccination status, and clinical risk factors, estimated VE was 24% (95% CI 5%–40%, *p =* 0.02). Full model parameters are available in S2 Table. Adjusted estimates of VE (aVE) were similar in patient subgroups restricted by (i) vaccine eligibility (*n =* 1,768, aVE 23%, 95% CI 1%–40%, *p =* 0.04) and (ii) age ≥ 65 years (*n =* 1,407, aVE 20%, 95% CI −5% to 40%, *p =* 0.11). In patients aged ≥75 years (*n =* 905), aVE was only 5% (95% CI −37% to 35%, *p =* 0.77). The mean times from vaccination to index admission with CAP for these patient subgroups were 10.4 (SD 5.4) years, 10.8 (SD 5.3) years, and 11.8 (SD 4.8) years, correspondingly.

**Table 2:** Unadjusted VE and aVE estimates.

	Cases *N* (%)	Controls *N* (%)	Unadjusted VE % (95% CI)	aVE % (95% CI)	*p*-Value Adjusted Analysis
***Primary Analysis*: *All PPV23 Serotypes***					
**Whole Cohort**					
Number	717	1,640			
Not vaccinated	373 (52.0)	746 (45.5)			
Vaccinated	344 (48.0)	894 (54.5)	**23 (8 to 35)**	**24 (5 to 40)**^a^	**0.02**
**Subgroup: Vaccine Eligible**					
Number	503	1,265			
Not vaccinated	189 (37.6)	416 (32.9)			
Vaccinated	314 (62.4)	849 (67.1)	19 (−1 to 34)	**23 (1 to 40)**^b^	**0.04**
**Subgroup: ≥65 Years**					
Number	414	993			
Not vaccinated	133 (32.1)	267 (26.9)			
Vaccinated	281 (67.9)	726 (73.1)	**22 (0 to 39)**	20 (−5 to 40)^c^	0.11
**Subgroup: ≥75 Years**					
Number	246	659			
Not vaccinated	65 (26.4)	168 (25.5)			
Vaccinated	181 (73.6)	491 (74.5)	5 (−33 to 32)	5 (−37 to 35)^d^	0.77
***Secondary Analysis***					
**PPV23/non-PCV13 Serotypes (Whole Cohort)**					
Number	417	1,640			
Not vaccinated	235 (56.4)	746 (45.5)			
Vaccinated	182 (43.7)	894 (54.5)	**35 (20 to 48)**	**29 (6 to 46)**^a^	**0.02**

^a^Adjusted for age, sex, receipt of seasonal flu vaccination, and presence or absence of the following risk factors: malignancy, cardiac failure, cerebrovascular disease, chronic renal disease, chronic liver disease, diabetes, ischaemic heart disease, COPD, other chronic cardiac disease, other chronic lung disease, hypertension, alcohol dependence, and immunosuppression.

^b^Adjusted for age, sex, receipt of seasonal flu vaccination.

^c^Adjusted for age group over 65, sex, receipt of seasonal flu vaccination, and presence or absence of a clinical risk factor.

^d^Adjusted for sex, receipt of seasonal flu vaccination, and presence or absence of a clinical risk factor only.

Unadjusted and adjusted results of the primary analysis, subgroup analysis, and the secondary case group analysis in cases against controls. The baseline group for all analysis is the respective control group. Vaccine exposure confirmed and self-reported yes at any point prior to their index admission. *p*-Values in bold are <0.05.

**Abbreviations:** aVE, adjusted VE; COPD, chronic obstructive pulmonary disease; PCV, pneumococcal conjugate vaccine; PPV23, 23-valent pneumococcal polysaccharide vaccine; VE, vaccine effectiveness

### Secondary analysis: PPV23/non-PCV13 cases and serotype specific

In the secondary analysis of PPV23/non-PCV13 serotype disease (*n =* 417, 43.7% vaccinated), the aVE was 29% (95% CI 6%–46%, *p =* 0.02) (Table 2). Similar estimates were observed in the vaccine-eligible (aVE 26%, 95% CI 0%–46%, *p =* 0.05) and ≥65-year-old (aVE 24%, 95% CI −7% to 47%, *p =* 0.12) subgroups. No vaccine effect was observed in the ≥75-year-old subgroup (aVE −2%, 95% CI −65% to 37%, *p =* 0.93). Serotype-specific aVE estimates varied by serotype. The highest estimates were seen in serotypes 3 (aVE 40%, 95% CI 14%–59%, *p =* 0.01), 12F (aVE 39%, 95% CI −20% to 69%, *p =* 0.15), 19F (aVE 38%, 95% CI −60% to 76%, *p =* 0.32), and 8 (aVE 34%, 95% CI 1%–55%, *p =* 0.04) (S3 Table). No vaccine effect was seen for serotypes 19A and 9N, while a negative aVE was observed for serotypes 5 (aVE −144%, 95% CI −503% to 1%, *p =* 0.05) and 11A (aVE −110%, 95% CI −415% to 14%, *p =* 0.1).

### Sensitivity analysis: Vaccine-confirmed cases

Patients with vaccine status confirmed through primary health records were older (67.9 versus 62.3 years) and more likely to have comorbid disease with higher severity disease on admission (24.2% versus 18.8% high severity disease) (S4 Table). A higher proportion were vaccinated with PPV23 (59.6% versus 28.7%). Sensitivity analysis of those with confirmed vaccine status produced slightly lower aVE estimates for all PPV23 cases (aVE 19%, 95% CI −5% to 37%, *p =* 0.12) and PPV23/non-PCV13 cases (aVE 21%, 95% CI −10% to 43%, *p =* 0.16) with confidence intervals crossing zero in both instances. There was no change in the primary outcome following sensitivity analysis of those missing both confirmed and self-reported vaccine status (*n =* 54).

### Time from vaccination

Data on date of vaccination were available for 535 (74.6%) cases of PPV23 serotype disease and 1,285 (78.3%) controls. The *p*-trend across time groups was 0.39, suggesting no association between time since vaccination and being a case (Table 3). For PPV23/non-PCV13 serotype disease, an association was observed (*p*-trend = 0.04); the highest aVE seen in those vaccinated within 5 years (aVE 46%) declining to 5% in those vaccinated ≥15 years prior (Table 3).

**Table 3:** Time from vaccination analysis.

	Number	Adjusted Odds Ratio	*p*-Trend	% aVE (95% CI)
**PPV23 Serotypes**				
Never	736	1		0
>15 years	228	0.9 (0.62 to 1.3)		10 (−30 to 38)
10–15 years	360	0.71 (0.51 to 0.99)		29 (1 to 49)
5–10 years	293	0.7 (0.49 to 0.98)		30 (2 to 51)
0–5 years	203	1.07 (0.74 to 1.54)	0.39	−7 (−54 to 26)
**PPV23/non-PCV13 Serotypes Only**				
Never	641	1		0
>15 years	196	0.95 (0.59 to 1.52)		5 (−52 to 41)
10–15 years	324	0.85 (0.56 to 1.29)		15 (−29 to 44)
5–10 years	264	0.81 (0.53 to 1.24)		19 (−24 to 47)
0–5 years	155	0.54 (0.31 to 0.95)	0.04	46 (5 to 69)

Adjusted odds ratios for case status between vaccinated and unvaccinated individuals in each time from vaccination group, compared to the baseline never vaccinated group. The *p*-trend across groups is presented. All estimates are adjusted for age, sex, receipt of seasonal flu vaccination, and presence or absence of the following risk factors: malignancy, cardiac failure, cerebrovascular disease, chronic renal disease, chronic liver disease, diabetes, ischaemic heart disease, COPD, other chronic cardiac disease, other chronic lung disease, hypertension, alcohol dependence, and immunosuppression. VE estimates are calculated as (1 –adjusted odds ratio) × 100.

**Abbreviations:** aVE, adjusted VE; COPD, chronic obstructive pulmonary disease; PCV, pneumococcal conjugate vaccine; PPV23, 23-valent pneumococcal polysaccharide vaccine; VE, vaccine effectiveness

**Fig 1:**
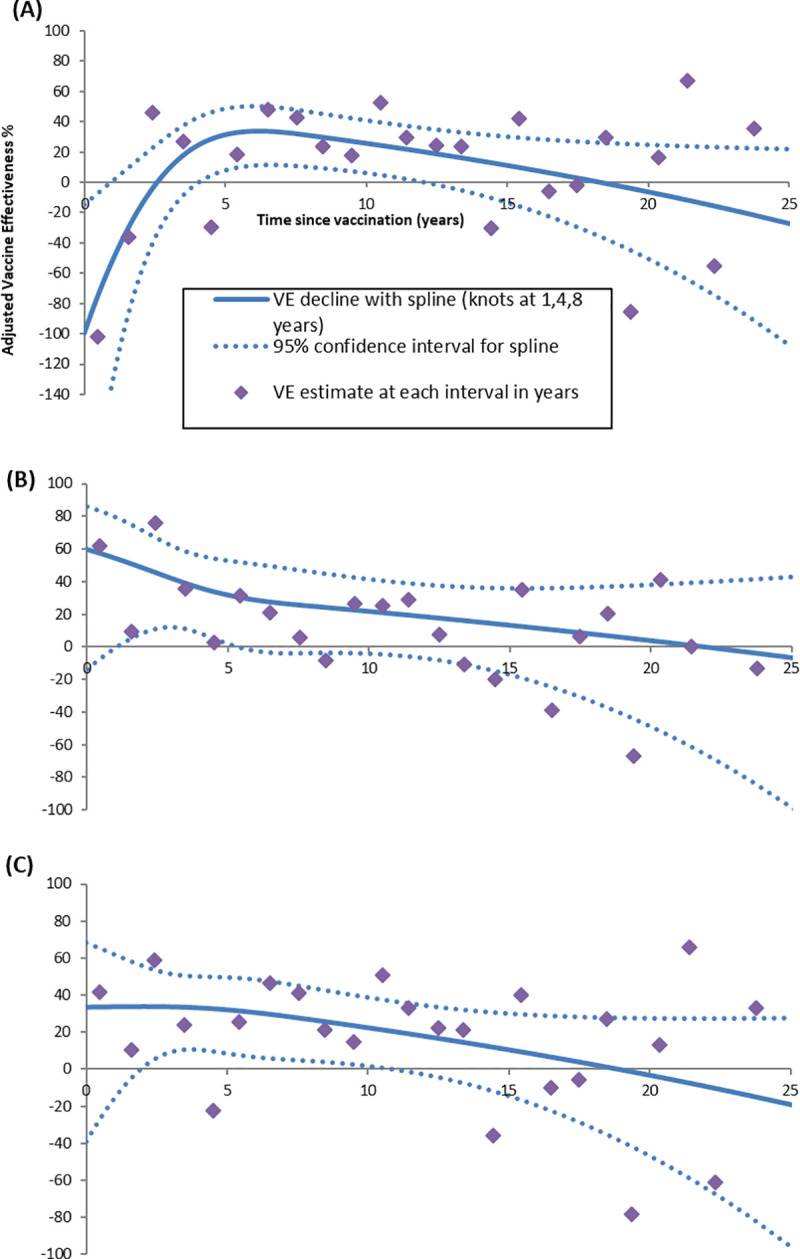
VE against time since vaccination using the spline model. VE by time since vaccination (in years) using the cubic spline model for the following case groups: (A) all PPV23 serotype disease, (B) PPV23/nonPCV13 serotype disease, and (C) all PPV23 serotype disease excluding serotype 5. Individual estimates for each year are shown but are based on small participant numbers within each year. PCV, pneumococcal conjugate vaccine; PPV23, 23-valent pneumococcal polysaccharide vaccine; VE, vaccine effectiveness.

A cubic spline model demonstrating change in VE with time since vaccination is shown in Fig 1. For PPV23 serotype disease, an inverted-U shape was observed suggesting a negative VE in those most recently vaccinated (Fig 1A). A post hoc descriptive analysis of cases vaccinated 0–5 years prior to admission found that the most commonly identified serotype was serotype 5 (*n =* 23, 34.6%) equating to 75.8% (23/33) of all serotype 5 cases. When serotype 5 cases were excluded from the PPV23 serotype case group, the inverted-U shape was not observed (Fig 1C).

## Discussion

The key study findings are that PPV23 vaccination provides moderate long-term protection in vaccinated individuals against hospitalisation with PPV23 serotype pneumonia (aVE 24%), with similar levels of protection evident for patient subgroups restricted to those who are vaccine eligible according to UK immunisation policy recommendations (aVE 23%) and patients aged ≥65 years (aVE 20%) but not for patients aged ≥75 years (aVE 5%). We also found protection against hospitalisation with PPV23/non-PCV13 serotype pneumonia to be similar (aVE 29%).

To our knowledge, only 2 other studies have previously reported on the serotype-specific effectiveness of PPV23 against pneumococcal pneumonia. Slightly higher VE estimates were reported by Suzuki and colleagues in their study of PPV23 effectiveness in adults over the age of 65 in Japan [14]. Using a similar test-negative design, they estimated PPV23 VE to be 33.5% (95% CI 5.6%–53.1%) against PPV23 serotypes and 27.4% (95% CI 3.2%–45.6%) against all pneumococcal pneumonia. There are differences that may account for our lower VE estimate. Firstly, our primary analysis included all vaccinated patients regardless of time of vaccination, whereas Suzuki and colleagues only considered a patient vaccinated if they had received the vaccine within 5 years prior to their index admission. Our estimates therefore represent long-term VE estimates. Secondly, our study took place in the setting of established PCV13 use within a strong national childhood vaccination program and resultant herd protection against these serotypes. In contrast, PCV13 replaced PCV7 in the Japanese childhood vaccination program only in the last 6 months of the study by Suzuki and colleagues.

A matched case-control study by Kim and colleagues of patients ≥65 years of age in the Republic of Korea found similar aVE estimates to ours in those aged 65–75 (aVE 21.0%) but no effect in those ≥75 years (aVE −35%) [21]. Of note, the median interval from vaccination to disease was short at 15 months, representing peak VE, and therefore their estimates are lower than might be expected. Both Suzuki and colleagues and Kim and colleagues relied on culture-based techniques of pneumococcal isolates to identify serotype [14,21]. As only a minority of patients with pneumococcal infection usually have positive respiratory and/or blood cultures, those studies represent a selected patient group [22].

Our VE estimates are lower than those reported for PPV23 vaccination against IPD. In a Cochrane review by Moberley and colleagues, the pooled odds of vaccination in cases of IPD was 0.26 (*n =* 11 randomised controlled trials [RCTs], 95% CI 0.14–0.45) and in vaccine-type IPD was 0.18 (*n =* 5 RCTs, 95% CI 0.1%–0.31%), equating to VE estimates of 74% and 82%, respectively [8]. As included clinical trials had shorter follow-up periods (2–3 years) compared to the mean time since vaccination observed in our study (10.4 years), their estimates likely represent maximal VE post-vaccination. In addition, Moberley and colleagues included older studies (pre-1970) that predominantly included cohorts of young, healthy individuals for whom the effect of immunosenescence is less important and consequently where VE estimates may be expected to be higher. Subgroup analysis by Moberley and colleagues of patients with known chronic disease from high-income countries found no protective effect, suggesting differential VE depending on underlying disease within IPD [8]. Using more recent UK data, Djennad and colleagues estimated VE of PPV23 against vaccine-type IPD in patients ≥65 years to be only slightly higher than those observed in our study of predominantly NIPP (IPD aVE 27%, 95% CI 17%–35%, bacteraemic pneumonia aVE 29%, 95% CI 17%–40%) [9]. Overall, it remains likely that PPV23 VE is greater against IPD than NIPP, but the size of difference may not be as large as previously estimated.

In our cohort of predominantly NIPP, we found the aVE for serotype 3 was 40% (95% CI 14%–59%). This is similar to VE estimates by Suzuki and colleagues (41.2%, 95% CI −10.8% to 68.8%) [14]. These results suggest that moderate direct protection is afforded by PPV23 against serotype 3 NIPP. Similar direct effects against serotype 3 NIPP in adults have been observed following PCV13 vaccination [23]. In contrast, in relation to adult IPD, Djennad and colleagues found no vaccine effect of PPV23 against serotype 3 [9]. Taken together, these results suggest that VE of PPV23 against serotype 3 may vary according to type of pneumococcal disease.

PPV23 induces an immune response via B cells in a time- and dose-dependent manner; due to its T-cell–independent mechanism, it is not expected to provide lifelong immunity via immunological memory [24]. The duration of protection afforded by the PPV23 vaccine is estimated at between 3 and 10 years [25,26]. Djennad and colleagues reported a decrease in VE estimates against PPV23 serotype disease ≥5 years since vaccination in their IPD cohort (0–2 years VE 41%; ≥5 years VE 23%) [9]. Our time interval analyses suggest a loss of protection in those vaccinated 10 to 15 years previously. This represents a longer durability of protection than might be expected from immunogenicity studies alone. However, our time-dependent estimates lack precision due to sample size limitations and may be affected by survival bias in those furthest from vaccination.

VE of PPV23 is known to differ by serotype within IPD [9]. In a post hoc analysis, we observed that serotype 5 was responsible for the largest proportion (34.3%) of cases of PPV23 serotype pneumonia within 0–5 years of vaccination. Since the introduction of PCV vaccines, serotype 5 has become an uncommon cause of IPD though it continues to be associated with cases of NIPP both in the UK and the US [6,27,28]. Prior to the introduction of PCV vaccines, it was considered a low-carriage, high-virulence serotype that could occur in disease outbreaks [29,30]. In our study, cases of serotype 5 pneumonia were spread evenly across the 5 years of the study, and we found no evidence for temporal clustering. Pimenta and colleagues recently reported other Streptococcal strains (*S*. *infantis*, *S*. *mitis*, and *S*. *oralis*) expressing serotype 5 capsule [31]. These pathogens commonly colonise the nasopharynx and mouth although they are not normally associated with a clinical diagnosis of CAP. The Bio-plex24 assay is highly sensitive [15]. It is therefore possible that there is a nonpneumococcal provenance for the detected serotype 5 antigen in our cases. However, such cross-reactivity would not in itself explain the differential effect in vaccinated and unvaccinated patients. We are not aware of any previous data, nor mechanism, to suggest that PPV23 vaccination might increase the risk of serotype 5 pneumonia and are currently unable to explain why 75% of cases of serotype 5 pneumonia occurred within 5 years of PPV23 vaccination in our study cohort. Accepting the possibility of a serotype 5 outbreak disproportionately affecting vaccinated individuals would mean that our estimates of PPV23 VE are conservative. Our observations around serotype 5 warrant further study, including confirmation in a separate cohort of patients.

### Strengths and limitations

The main strengths of this study are (i) the use of a serotype-specific multiplex urine assay allowing analysis of VE in both IPD and NIPP across all serotypes included within the PPV23 vaccine, (ii) analysis based on a large cohort of consecutively consented patients without knowledge of the causative serotype at the time of recruitment, thereby minimising selection bias, and (iii) findings set in the background of a strong national PCV13 childhood vaccination programme providing well-established adult herd protection effects. Vaccine status, including date of vaccine, was confirmed through primary care records in a high proportion of patients, thus minimising the effect of recall and misclassification bias. The accuracy of those with self-reported and confirmed vaccine status was 82.7% for those who self-reported as ‘vaccinated’ and 56.1% for those who self-reported as ‘not vaccinated’; the direction of bias when including self-reported vaccine status is therefore towards a more conservative VE estimate [32].

The study is subject to the inherent biases common to case-control studies; however, the main limitation is lack of power. Due to relatively high vaccination rates in both case and control groups, our analysis is underpowered to reject the null hypothesis (that there is no vaccine effect observed); 2,100 patients would be required in each outcome group for 80% power at a significance level of 0.05 for a VE of 22%, estimated on the vaccine exposure within the whole cohort. The statistically significant results observed therefore are likely to represent true findings. However, the study sample was not large enough to enable robust subgroup analyses of VE by age groups above 65 years. Secondly, of those identified as eligible for the cohort study on which this analysis was conducted, patients in whom study consent was not obtained were older (median age 82.2 years) with more comorbid disease [6]. Therefore, VE estimates presented here may be less applicable to persons aged above 80 years [33]. Due to the retrospective nature of the study, adjusting by time since vaccination is not possible in the unvaccinated cohort. Our case group were more likely to be female. Close contact with children has previously been found to be associated with an increased risk of pneumococcal disease [34,35]. The observed female predominance in cases may reflect sex differences in level of close contact with children.

### Implications

This study suggests that single-dose adult PPV23 vaccination in the setting of an established childhood PCV13 vaccination programme provides moderate VE against hospitalisation with PPV23 serotype pneumonia and that the current UK adult pneumococcal vaccine policy appropriately identifies clinical at-risk patient groups who benefit from PPV23 vaccination. However, there is a suggestion, consistent with data from other studies, that protection more than 15 years after vaccination is low. In many countries, the vast majority of adults who receive PPV23 vaccination do so at, or before, the age of 65 years, while the median age of adults hospitalised with CAP is around 75 years [36]. This raises questions regarding the timing of adult pneumococcal vaccination and the role and value of revaccination in the context of an ageing population [37,38]. Repeat vaccination with PPV23 has been found to safely produce immunogenic antibody responses with limited evidence of immune hypo-responsiveness following an interval of 5 years or more; however, studies in high-risk populations have not been able to show VE against IPD following revaccination [39,40]. Newer multivalent PCV vaccines are coming to market and may provide alternative options to consider.

### Conclusions

In the setting of an established national childhood PCV13 vaccination programme, PPV23 vaccination in clinical at-risk patient groups and adults ≥65 years of age appears moderately effective against hospitalisation with PPV23 serotype pneumococcal pneumonia.

## Supporting information

S1 Text STROBE checklist. STROBE, Strengthening the Reporting of Observational Studies in Epidemiology.
(DOC)

S2 Text Prospective statistical analysis plan—March 2019. (DOCX)

S1 Table Characteristics of the unvaccinated and vaccinated patient cohorts. Unadjusted odds ratios with 95% CIs are presented with *p*-values. The baseline group for all analysis is the unvaccinated cohort. *p-Trend derived from chi-squared test for trend.
(DOCX)

S2 Table Estimated model parameters for the primary analysis model. Odds ratios, 95% CIs, and *p*-values are displayed.
(DOCX)

S3 Table Unadjusted and adjusted results of the serotype-specific analysis. The baseline group for all analysis is the respective control group. Vaccine exposure confirmed and self-reported yes at any point prior to their index admission. Results are adjusted for age, sex, receipt of seasonal flu vaccination, and presence or absence of a clinical risk factor only.
(DOCX)

S4 Table Characteristics of patients in whom the vaccine status was confirmed via primary care compared to those with self-reports only. *p-Trend derived from chi-squared test for trend.
(DOCX)

S5 Table Subanalysis of the cohort restricted to 60- to 75-year-olds with no known risk factors. Unadjusted and adjusted results of the primary analysis and the secondary case group analysis in cases against controls. The baseline group for all analysis is the respective control group. Vaccine exposure confirmed and self-reported yes at any point prior to their index admission. Adjusted for sex only.
(DOCX)

S6 Table Subanalysis excluding serotype 5 from the primary analysis group (all PPV23 serotypes). Adjusted for age, sex, receipt of seasonal flu vaccination, and presence or absence of the following risk factors: malignancy, cardiac failure, cerebrovascular disease, chronic renal disease, chronic liver disease, diabetes, ischaemic heart disease, COPD, other chronic cardiac disease, other chronic lung disease, hypertension, alcohol dependence, and immunosuppression. ^Adjusted for age, sex, receipt of seasonal flu vaccination. COPD, chronic obstructive pulmonary disease; PPV23, 23-valent pneumococcal polysaccharide vaccine.
(DOCX)

## Abbreviations

aVE: adjusted VE
CAP: community-acquired pneumonia
COPD: chronic obstructive pulmonary disease
CPS: common polysaccharide
IPD: invasive pneumococcal disease
IQR: interquartile range
NIPP: non-invasive pneumococcal pneumonia
PCV: pneumococcal conjugate vaccine
PPV23: 23-valent pneumococcal polysaccharide vaccine
RCT: randomised controlled trials
REC: Research Ethics Committee
STROBE: Strengthening the Reporting of Observational Studies in Epidemiology
VE: vaccine effectiveness

## References

[1] Said MA, Johnson HL, Nonyane BA, Deloria-Knoll M, O'Brien KL, Andreo F, et al Estimating the burden of pneumococcal pneumonia among adults: a systematic review and meta-analysis of diagnostic techniques. PLoS ONE. 2013;8(4):e60273 Epub 2013/04/09. 10.1371/journal.pone.0060273 23565216 PMC3615022

[2] Welte T, Torres A, Nathwani D. Clinical and economic burden of community-acquired pneumonia among adults in Europe. Thorax. 2012;67(1):71–9. Epub 2010/08/24. 10.1136/thx.2009.129502 . 20729232

[3] Public Health England P. Immunisation against infectious disease—The Green Book. Chapter 25: Pneumococcal 2018 [cited 2019 Dec 1]. Available from: https://assets.publishing.service.gov.uk/government/uploads/system/uploads/attachment_data/file/674074/GB_Chapter_25_Pneumococcal_V7_0.pdf.

[4] Kandasamy R, Voysey M, Collins S, Berbers G, Robinson H, Noel I, et al Persistent Circulation of Vaccine Serotypes and Serotype Replacement After 5 Years of Infant Immunization With 13-Valent Pneumococcal Conjugate Vaccine in the United Kingdom. The Journal of infectious diseases. 2019 10.1093/infdis/jiz178 31004136

[5] Ladhani SN, Collins S, Djennad A, Sheppard CL, Borrow R, Fry NK, et al Rapid increase in non-vaccine serotypes causing invasive pneumococcal disease in England and Wales, 2000–17: a prospective national observational cohort study. The Lancet Infectious diseases. 2018;18(4):441–51. Epub 2018/02/06. 10.1016/S1473-3099(18)30052-5 . 29395999

[6] Pick H, Daniel P, Rodrigo C, Bewick T, Ashton D, Lawrence H, et al Pneumococcal serotype trends, surveillance and risk factors in UK adult pneumonia, 2013–18. Thorax. 2019 Epub 2019/10/09. 10.1136/thoraxjnl-2019-213725 . 31594801

[7] Public Health England P. Pneumococcal Polysacchardie Vaccine (PPV) coverage report, England, April 2017 to March 2018. 2018 27 July 2018. Report No.: Number 27.

[8] Moberley S, Holden J, Tatham DP, Andrews RM. Vaccines for preventing pneumococcal infection in adults. The Cochrane database of systematic reviews. 2013;(1):Cd000422 Epub 2013/02/27. 10.1002/14651858.CD000422.pub3 . 23440780 PMC7045867

[9] Djennad A, Ramsay ME, Pebody R, Fry NK, Sheppard C, Ladhani SN, et al Effectiveness of 23-Valent Polysaccharide Pneumococcal Vaccine and Changes in Invasive Pneumococcal Disease Incidence from 2000 to 2017 in Those Aged 65 and Over in England and Wales. EClinicalMedicine. 2018;6:42–50. Epub 2019/06/14. 10.1016/j.eclinm.2018.12.007 31193709 PMC6537583

[10] Huss A, Scott P, Stuck AE, Trotter C, Egger M. Efficacy of pneumococcal vaccination in adults: a meta-analysis. Cmaj. 2009;180(1):48–58. Epub 2009/01/07. 10.1503/cmaj.080734 19124790 PMC2612051

[11] Thorrington D, Andrews N, Stowe J, Miller E, van Hoek AJ. Elucidating the impact of the pneumococcal conjugate vaccine programme on pneumonia, sepsis and otitis media hospital admissions in England using a composite control. BMC medicine. 2018;16(1):13 Epub 2018/02/09. 10.1186/s12916-018-1004-z 29415741 PMC5804014

[12] Rodrigo C, Bewick T, Sheppard C, Greenwood S, McKeever TM, Trotter CL, et al Impact of infant 13-valent pneumococcal conjugate vaccine on serotypes in adult pneumonia. The European respiratory journal. 2015;45(6):1632–41. Epub 2015/03/21. 10.1183/09031936.00183614 . 25792633

[13] Boddington NL, Warburton F, Zhao H, Andrews N, Ellis J, Donati M, et al Influenza vaccine effectiveness against hospitalisation due to laboratory-confirmed influenza in children in England in the 2015–2016 influenza season—a test-negative case-control study. Epidemiology and infection. 2019;147:e201-e 10.1017/S0950268819000876 . 31364557 PMC6624859

[14] Suzuki M, Dhoubhadel BG, Ishifuji T, Yasunami M, Yaegashi M, Asoh N, et al Serotype-specific effectiveness of 23-valent pneumococcal polysaccharide vaccine against pneumococcal pneumonia in adults aged 65 years or older: a multicentre, prospective, test-negative design study. The Lancet Infectious diseases. 2017;17(3):313–21. Epub 2017/01/28. 10.1016/S1473-3099(17)30049-X . 28126327

[15] Eletu SD, Sheppard CL, Thomas E, Smith K, Daniel P, Litt DJ, et al Development of an Extended-Specificity Multiplex Immunoassay for Detection of Streptococcus pneumoniae Serotype-Specific Antigen in Urine by Use of Human Monoclonal Antibodies. Clin Vaccine Immunol. 2017;24(12). Epub 2017/10/06. 10.1128/CVI.00262-17 28978509 PMC5717182

[16] Eletu SD, Sheppard CL, Rose S, Smith K, Andrews N, Lim WS, et al Re-validation and update of an extended-specificity multiplex assay for detection of Streptococcus pneumoniae capsular serotype/serogroup-specific antigen and cell-wall polysaccharide in urine specimens. Access Microbiology. 2020 10.1099/acmi.0.000094. PMC7470314 32974571

[17] Oken MM, Creech RH, Tormey DC, Horton J, Davis TE, McFadden ET, et al Toxicity and response criteria of the Eastern Cooperative Oncology Group. American journal of clinical oncology. 1982;5(6):649–55. Epub 1982/12/01. . 7165009

[18] Textor J, Hardt J, Knuppel S. DAGitty: a graphical tool for analyzing causal diagrams. Epidemiology (Cambridge, Mass). 2011;22(5):745 Epub 2011/08/04. 10.1097/EDE.0b013e318225c2be . 21811114

[19] Sintes X, Nebot M, Izquierdo C, Ruiz L, DomÍNguez A, Bayas JM, et al Factors associated with pneumococcal and influenza vaccination in hospitalized people aged ⩾65 years. Epidemiology and infection. 2011;139(5):666–73. Epub 2010/08/09. 10.1017/S0950268810001846 20696084

[20] StataCorp. Stata Statistical Software: Release 16. College Station, TX: StataCorp LLC 2019.

[21] Kim JH, Chun BC, Song JY, Kim HY, Bae IG, Kim DM, et al Direct effectiveness of pneumococcal polysaccharide vaccine against invasive pneumococcal disease and non-bacteremic pneumococcal pneumonia in elderly population in the era of pneumococcal conjugate vaccine: A case-control study. Vaccine. 2019;37(21):2797–804. Epub 2019/04/22. 10.1016/j.vaccine.2019.04.017 . 31005428

[22] Haessler S, Lindenauer PK, Zilberberg MD, Imrey PB, Yu PC, Higgins T, et al Blood cultures versus respiratory cultures: Two different views of pneumonia. Clinical infectious diseases: an official publication of the Infectious Diseases Society of America. 2019 Epub 2019/10/31. 10.1093/cid/ciz1049 . 31665249 PMC7882190

[23] McLaughlin JM, Jiang Q, Gessner BD, Swerdlow DL, Sings HL, Isturiz RE, et al Pneumococcal conjugate vaccine against serotype 3 pneumococcal pneumonia in adults: A systematic review and pooled analysis. Vaccine. 2019;37(43):6310–6. Epub 2019/09/17. 10.1016/j.vaccine.2019.08.059 . 31522807

[24] Vadlamudi NK, Parhar K, Altre Malana KL, Kang A, Marra F. Immunogenicity and safety of the 13-valent pneumococcal conjugate vaccine compared to 23-valent pneumococcal polysaccharide in immunocompetent adults: A systematic review and meta-analysis. Vaccine. 2019;37(8):1021–9. 10.1016/j.vaccine.2019.01.014 30685252

[25] Sankilampi U, Honkanen PO, Bloigu A, Leinonen M. Persistence of antibodies to pneumococcal capsular polysaccharide vaccine in the elderly. The Journal of infectious diseases. 1997;176(4):1100–4. Epub 1997/10/23. 9333177. 10.1086/516521 9333177

[26] Vaccines against influenza WHO position paper—November 2012. Releve epidemiologique hebdomadaire. 2012;87(47):461–76. Epub 2012/12/06. . 23210147

[27] Sherwin RL, Gray S, Alexander R, McGovern PC, Graepel J, Pride MW, et al Distribution of 13-valent pneumococcal conjugate vaccine Streptococcus pneumoniae serotypes in US adults aged > = 50 years with community-acquired pneumonia. Journal of Infectious Diseases. 2013;208(11):1813–20. 10.1093/infdis/jit506 . 24092845

[28] Isturiz RE, Ramirez J, Self WH, Grijalva CG, Counselman FL, Volturo G, et al Pneumococcal epidemiology among us adults hospitalized for community-acquired pneumonia. Vaccine. 2019;37(25):3352–61. Epub 2019/05/11. 10.1016/j.vaccine.2019.04.087 . 31072732

[29] Hausdorff WP, Feikin DR, Klugman KP. Epidemiological differences among pneumococcal serotypes. The Lancet Infectious diseases. 2005;5(2):83–93. Epub 2005/02/01. 10.1016/S1473-3099(05)01280-6 . 15680778

[30] Romney MG, Hull MW, Gustafson R, Sandhu J, Champagne S, Wong T, et al Large community outbreak of Streptococcus pneumoniae serotype 5 invasive infection in an impoverished, urban population. Clinical infectious diseases: an official publication of the Infectious Diseases Society of America. 2008;47(6):768–74. Epub 2008/08/12. 10.1086/591128 . 18690803

[31] Pimenta F, Gertz RE Jr., Park SH, Kim E, Moura I, Milucky J, et al Streptococcus infantis, Streptococcus mitis, and Streptococcus oralis Strains With Highly Similar cps5 Loci and Antigenic Relatedness to Serotype 5 Pneumococci. Frontiers in microbiology. 2018;9:3199 Epub 2019/01/24. 10.3389/fmicb.2018.03199 30671034 PMC6332807

[32] Jackson ML. Use of self-reported vaccination status can bias vaccine effectiveness estimates from test-negative studies. Vaccine: X. 2019;1:100003 10.1016/j.jvacx.2018.100003. PMC6668222 33826685

[33] Ciabattini A, Nardini C, Santoro F, Garagnani P, Franceschi C, Medaglini D. Vaccination in the elderly: The challenge of immune changes with aging. Seminars in Immunology. 2018;40:83–94. 10.1016/j.smim.2018.10.010 30501873

[34] Daniel P, Rodrigo C, Bewick T, Sheppard C, Greenwood S, McKeever TM, et al Increased incidence of adult pneumococcal pneumonia during school holiday periods. ERJ open research. 2017;3(1). Epub 2017/03/23. 10.1183/23120541.00100–2016 28326311 PMC5349095

[35] Rodrigo C, Bewick T, Sheppard C, Greenwood S, Macgregor V, Trotter C, et al Pneumococcal serotypes in adult non-invasive and invasive pneumonia in relation to child contact and child vaccination status. Thorax. 2014;69(2):168–73. Epub 2013/09/21. 10.1136/thoraxjnl-2013-203987 . 24048505

[36] British Thoracic Society B, Lawrence H, Lim WS. National Audit Report: Adult Community Acquired Pneumonia Audit 2018–2019. Online: 2019.

[37] Robert Koch Institut S. Recommentations of the Standing Committee on Vacciantion (STIKO) at the Robert Koch Institue—2017/2018. 2017:Page 371.

[38] Thorrington D, van Rossum L, Knol M, de Melker H, Rumke H, Hak E, et al Impact and cost-effectiveness of different vaccination strategies to reduce the burden of pneumococcal disease among elderly in the Netherlands. PLoS ONE. 2018;13(2):e0192640 Epub 2018/02/10. 10.1371/journal.pone.0192640 29425249 PMC5806887

[39] Caya CA, Boikos C, Desai S, Quach C. Dosing regimen of the 23-valent pneumococcal vaccination: a systematic review. Vaccine. 2015;33(11):1302–12. Epub 2015/02/11. 10.1016/j.vaccine.2015.01.060 . 25660650

[40] Takashima M, Lambert SB, Paynter S, Ware RS. Relative effectiveness of revaccination with 23-valent pneumococcal polysaccharide vaccine in preventing invasive pneumococcal disease in adult Aboriginal and Torres Strait Islander people, Australia. Vaccine. 2019;37(12):1638–41. Epub 2019/02/21. 10.1016/j.vaccine.2019.01.085 . 30782489

