# Supplementary material for: Effectiveness of the 23-valent pneumococcal polysaccharide vaccine against vaccine serotype pneumococcal pneumonia in adults: A case-control test-negative design study
Source: PLoS Med. 2020 Oct 23;17(10):e1003326. doi: 10.1371/journal.pmed.1003326 (PMC7584218; doi:10.1371/journal.pmed.1003326)
Supplement: S2 Text — (DOCX) [file pmed.1003326.s002.docx]

# PPV23 Vaccine Effectiveness Study – Analysis Plan

## Study Aims:

To evaluate the effectiveness of the PPV23 vaccine in against PPV23 vaccine-type pneumococcal pneumonia in adults hospitalise with community-acquired pneumonia (CAP).

Secondary aims:

- To estimate vaccine effectiveness (VE) in patient sub-groups based on age and clinical risk.
- To estimate the VE against PPV23/non-PCV13 pneumonia.
- To examine the effect of time since vaccination on VE, if able.

## Methods:

### Study Design

Retrospective modified case-control study using a test negative design.

### Cohort:

The cohort consists of SCAPA study participants (years 6-10 inclusive) with urine samples subjected to testing for pneumococcal disease using the Bio-plex24 assay. Pneumococcal serotype is confirmed by: i) Bioplex testing of urine samples or ii) blood culture testing for *Strep. pneumoniae* isolates with subsequent serotyping.

### Definition of Cases and Controls:

#### Controls

A control patient is defined as non-PPV23 vaccine serotype pneumococcal disease or non-pneumococcal pneumonia. This includes:

- Bioplex negative cases (pneumonia of alternate unknown aetiology)
- Non-PPV23 serotype Bioplex positive cases
- Bioplex positive with capsular protein but no serotype identified
- Pneumonia with a confirmed alternative aetiology (via legionella urinary antigen testing or blood culture).

#### Cases - Serotype Groups

A case of PPV23 disease was defined as pneumococcal pneumonia with the following causative serotypes contained within the PPV23 vaccine: 1, 2, 3, 4, 5, 6B, 7F, 8, 9V/N, 10A, 11A, 12F, 14, 15B, 17F, 18C, 19A, 19F, 20, 22F, 23F, 33F.

The secondary case groups for analysis is PPV23/non-PCV13 serotypes (1, 3, 4, 5, **6B**, 7F, 9V, 14, 18C, 19A, 19F, 23F). Serotype 6A is excluded as it is not PPV23 vaccine serotype. Serotype specific analysis to be performed if case numbers allow.

#### Multiple Serotypes

Where multiple serotypes are identified in a single case it is not possible to say which serotype is causative. These patients are excluded only if the identified serotypes cross serotype groupings. If all identified serotypes are within one group, the case is included within that group.

### Vaccine status:

The primary analysis includes all adults, regardless of vaccine eligibility. In the UK, adults ≥65 or <65 years with a clinical risk factor as per the Green Book are considered vaccine eligible. Further subgroup analyses to include patients who are: i) vaccine eligible under current UK pneumococcal vaccine policy, ii) aged ≥65 years, and iii) aged ≥75 at the time of their index admission.

Vaccine status is self-reported by the participant during recruitment and confirmed through electronic records where available. All recruits without confirmed vaccine dates to be excluded from vaccine time interval analysis.

Sub-analyses to be performed using recruits who were i) vaccinated ever and ii) vaccinated within 3, 5 and 10 years of their index admission. The control group for each vaccine timeframe to be the never vaccinated group.

No matching of cases with controls to be conducted.

### Statistical Plan:

Descriptive statistics comparing the exposed and the unexposed groups to be calculated using the appropriate summary statistic for the variable (proportions for binary variables, median & IQR for non-parametric continuous variables). Odds ratios with 95% confidence intervals and p values for significance testing calculated for binary variables. A p value of <0.05 considered significant. Hypothesis testing for non-parametric variables performed using Mann Witney U test. Likelihood ratio tests for hypothesis testing of associations between the exposure group and ordered categorical variables (eg. Baseline performance status and CURB65 severity category).

Multiple logistic regression models to derive adjusted odds ratios of being a case in the vaccinated vs unvaccinated groups. Confounders included in the model as a priori: age at index admission, gender, clinical risk groups as defined by the green book and flu vaccination status. A minimum adjustment variable set of confounders to be identified using a Directed Acyclic Graph (DAG). Vaccine effectiveness estimates to be calculated as (1-odds ratio) x 100%. Analysis performed using STATA 16©.
