# Supplementary material for: Effectiveness of the 23-valent pneumococcal polysaccharide vaccine against vaccine serotype pneumococcal pneumonia in adults: A case-control test-negative design study
Source: PLoS Med. 2020 Oct 23;17(10):e1003326. doi: 10.1371/journal.pmed.1003326 (PMC7584218; doi:10.1371/journal.pmed.1003326)
Supplement: S1 Table — Unadjusted odds ratios with 95% CIs are presented with p-values. The baseline group for all analysis is the unvaccinated cohort. *p-Trend derived from chi-squared test for trend. (DOCX) [file pmed.1003326.s003.docx]

### S1 Table: Characteristics of the Unvaccinated and Vaccinated patient cohorts

|  | **Unvaccinated patients** | **Vaccinated patients** | **Odds Ratio** | **95% Confidence Interval** | **p-value** |
| --- | --- | --- | --- | --- | --- |
| Number | 1119 | 1238 |  |  |  |
| Mean Age (SD) | 57.4 (19.0) | 74.1 (13.5) | 1.05 | 1.05-1.06 | **<0.0001** |
| Sex (male %) | 619 (55.3) | 654 (52.8) | 1.01 | 0.93-1.29 | 0.26 |
| Residential care | 30 (2.7) | 51 (4.1) | 1.56 | 0.98-2.47 | 0.06 |
| Baseline PS (%): | | | | | |
| 0 | 480 (42.9) | 327 (26.4) | 1 |  |  |
| 1 | 351 (31.4) | 465 (37.6) | 1.94 | 1.60-2.37 |  |
| 2 | 155 (13.9) | 259 (20.9) | 2.45 | 1.92-3.13 |  |
| 3 | 49 (4.4) | 63 (5.1) | 1.89 | 1.27-2.81 |  |
| 4 | 36 (3.2) | 35 (2.8) | 1.43 | 0.88-2.32 | **<0.0001*** |
| Missing | 48 (4.3) | 89 (7.2) |  |  |  |
| **Severity (%):** | | | | | |
| Low | 695 (62.1) | 438 (35.4) | 1 |  |  |
| Moderate | 251 (22.4) | 432 (34.9) | 2.73 | 2.24-3.32 |  |
| Severe | 173 (15.5) | 368 (29.7) | 3.38 | 2.72-4.19 | **<0.0001*** |
| **Co-morbidities:** | | | | | |
| Malignancy | 80 (7.2) | 140 (11.3) | 1.65 | 1.24-2.20 | 0.0006 |
| Liver Disease | 31 (2.8) | 19 (1.5) | 0.55 | 0.31-0.97 | 0.04 |
| CCF | 45 (4.0) | 100 (8.1) | 2.1 | 1.46-3.01 | <0.0001 |
| CVA | 45 (4.0) | 134 (10.8) | 2.9 | 2.04-4.11 | <0.0001 |
| Renal disease | 68 (6.1) | 156 (12.6) | 2.22 | 1.65-3.01 | <0.0001 |
| Diabetes | 122 (10.9) | 254 (20.5) | 2.11 | 1.67-2.67 | <0.0001 |
| IHD | 73 (6.5) | 178 (14.4) | 2.41 | 1.80-3.21 | <0.0001 |
| Cognitive impairment | 23 (2.1) | 58 (4.7) | 2.34 | 1.43-3.83 | 0.0005 |
| Asthma | 130 (11.6) | 117 (9.5) | 0.79 | 0.61-1.03 | 0.09 |
| COPD | 158 (14.1) | 404 (32.6) | 2.95 | 2.39-3.64 | <0.0001 |
| Chronic heart disease | 110 (9.8) | 253 (20.4) | 2.36 | 1.85-3.00 | <0.0001 |
| Chronic lung disease | 184 (16.4) | 454 (36.7) | 2.94 | 2.41-3.60 | <0.0001 |
| Hypertension | 193 (17.3) | 378 (30.5) | 2.11 | 1.73-2.57 | <0.0001 |
| Alcohol | 42 (3.8) | 18 (1.5) | 0.38 | 0.22-0.66 | 0.0004 |
| Immunosuppression | 42 (3.7) | 66 (5.3) | 1.44 | 0.97-2.15 | 0.07 |

**S1 Table**: Characteristics of the unvaccinated and vaccinated patient cohorts. Unadjusted odds ratios with 95% confidence intervals are presented with p values. The baseline group for all analysis is the unvaccinated cohort. *p-value for trend derived from chi-squared test for trend
