# Supplementary material for: Effectiveness of the 23-valent pneumococcal polysaccharide vaccine against vaccine serotype pneumococcal pneumonia in adults: A case-control test-negative design study
Source: PLoS Med. 2020 Oct 23;17(10):e1003326. doi: 10.1371/journal.pmed.1003326 (PMC7584218; doi:10.1371/journal.pmed.1003326)
Supplement: S2 Table — Odds ratios, 95% CIs, and p-values are displayed. (DOCX) [file pmed.1003326.s004.docx]

### S2 Table: Estimated model parameters for the primary analysis

|  | **OR** | **95% CI** | **P value** |
| --- | --- | --- | --- |
| **Previous PPV23 vaccination with PPV23** | 0.76 | 0.6-0.95 | 0.02 |
|  | | | |
| **Age** | 1 | 0.99-1.01 | 0.51 |
| **Sex (male)** | 0.68 | 0.57-0.82 | <0.0001 |
| **Seasonal influenza vaccination** | 1.01 | 0.81-1.25 | 0.96 |
| **Risk factors:** | | | |
| Cancer | 0.7 | 0.5-0.99 | 0.04 |
| Cardiac failure | 1.2 | 0.58-2.48 | 0.63 |
| Cerebrovascular disease | 1.05 | 0.74-1.5 | 0.77 |
| Renal disease | 1.05 | 0.76-1.44 | 0.78 |
| Liver disease | 1.17 | 0.62-2.21 | 0.62 |
| Diabetes | 0.95 | 0.73-1.22 | 0.67 |
| Ischaemic heart disease | 1.55 | 0.71-3.4 | 0.27 |
| Cognitive impairment | 1.12 | 0.69-1.82 | 0.66 |
| Chronic obstructive pulmonary disease | 1.05 | 0.62-1.78 | 0.86 |
| Chronic lung disease | 1.01 | 0.61-1.68 | 0.97 |
| Hypertension | 1.15 | 0.92-1.43 | 0.21 |
| Alcohol excess | 1.84 | 1.06-3.21 | 0.03 |
| Immunosuppression | 1.13 | 0.73-1.75 | 0.59 |

**S2 Table:** Estimated model parameters for the primary analysis model. Odds ratios, 95% confidence intervals and p-values are displayed.
