# Supplementary material for: Effectiveness of the 23-valent pneumococcal polysaccharide vaccine against vaccine serotype pneumococcal pneumonia in adults: A case-control test-negative design study
Source: PLoS Med. 2020 Oct 23;17(10):e1003326. doi: 10.1371/journal.pmed.1003326 (PMC7584218; doi:10.1371/journal.pmed.1003326)
Supplement: S3 Table — The baseline group for all analysis is the respective control group. Vaccine exposure confirmed and self-reported yes at any point prior to their index admission. Results are adjusted for age, sex, receipt of seasonal flu vaccination, and presence or absence of a clinical risk factor only. (DOCX) [file pmed.1003326.s005.docx]

### S3 Table: Serotype Specific Analysis

|  | **Cases N (%)** | **Controls N (%)** | **Unadjusted Vaccine Effectiveness % (95% CI)** | **Adjusted Vaccine Effectiveness % (95% CI)** | **p-value adjusted analysis** |
| --- | --- | --- | --- | --- | --- |
| **Serotype Specific Analysis** | | | | | |
| **Serotype 3** | | | | | |
| Number | 197 | 1640 |  |  |  |
| Not vaccinated | 96 (48.7) | 746 (45.5) |  |  |  |
| Vaccinated | 101 (51.3) | 894 (54.5) | 12 (-18 to 35) | **40 (14 to 59)** | **0.01** |
| **Serotype 8** | | | | | |
| Number | 192 | 1640 |  |  |  |
| Not vaccinated | 119 (62.0) | 746 (45.5) |  |  |  |
| Vaccinated | 73 (38.0) | 894 (54.5) | **49 (30 to 62)** | **34 (1 to 55)** | **0.04** |
| **Serotype 12F** | | | | | |
| Number | 60 | 1640 |  |  |  |
| Not vaccinated | 39 (65.0) | 746 (45.5) |  |  |  |
| Vaccinated | 21 (35.0) | 894 (54.5) | **55 (23 to 74)** | **39 (-20 to 69)** | **0.2** |
| **Serotype 5** | | | | | |
| Number | 41 | 1640 |  |  |  |
| Not vaccinated | 9 (22.0) | 746 (45.5) |  |  |  |
| Vaccinated | 32 (78.0) | 894 (54.5) | **-197 (-527 to -40)** | -144 (-503 to 1) | 0.05 |
| **Serotype 11A** | | | | | |
| Number | 36 | 1640 |  |  |  |
| Not vaccinated | 11 (30.6) | 746 (45.5) |  |  |  |
| Vaccinated | 25 (69.4) | 894 (54.5) | -90 (-288 to 7) | -110 (-415 to 14) | 0.1 |
| **Serotype 19A** | | | | | |
| Number | 34 | 1640 |  |  |  |
| Not vaccinated | 17 (50.0) | 746 (45.5) |  |  |  |
| Vaccinated | 17 (50.0) | 894 (54.5) | 17 (-65 to 58) | 14 (-105 to 64) | 0.74 |
| **Serotype 9N** | | | | | |
| Number | 32 | 1640 |  |  |  |
| Not vaccinated | 13 (40.6) | 746 (45.5) |  |  |  |
| Vaccinated | 19 (59.4) | 894 (54.5) | -22 (-150 to 40) | -15 (-173 to 52) | 0.75 |
| **Serotype 19F** | | | | | |
| Number | 25 | 1640 |  |  |  |
| Not vaccinated | 10 (40.0) | 746 (45.5) |  |  |  |
| Vaccinated | 15 (60.0) | 894 (54.5) | -25 (-180 to 44) | 38 (-60 to 76) | 0.32 |

**S3 Table:** Unadjusted and adjusted results of the serotype specific analysis. The baseline group for all analysis is the respective control group. Vaccine exposure confirmed and self-reported yes at any point prior to their index admission. Results are adjusted for age, gender, receipt of seasonal flu vaccination and presence or absence of a clinical risk factor only.
