# Supplementary material for: Effectiveness of the 23-valent pneumococcal polysaccharide vaccine against vaccine serotype pneumococcal pneumonia in adults: A case-control test-negative design study
Source: PLoS Med. 2020 Oct 23;17(10):e1003326. doi: 10.1371/journal.pmed.1003326 (PMC7584218; doi:10.1371/journal.pmed.1003326)
Supplement: S4 Table — *p-Trend derived from chi-squared test for trend. (DOCX) [file pmed.1003326.s006.docx]

### S4 Table: Characteristics of vaccine confirmed vs self-reported only patients

|  | **Vaccine status confirmed through medical records**  **N (%)** | **Self-reported vaccine status only**  **N (%)** | **p-value** |
| --- | --- | --- | --- |
| Number | 1820 | 537 |  |
| Mean Age (SD) | 67.9 (17.8) | 62.3 (19.3) | **<0.0001** |
| Gender (male) | 971 (53.3) | 303 (56.4) | 0.21 |
| Residential care | 70 (3.8) | 11 (2.1) | 0.06 |
| Vaccinated | 1084 (59.6) | 154 (28.7) | **<0.0001** |
| **Baseline Performance Status:** | | | |
| 0 | 605 (33.2) | 202 (37.6) |  |
| 1 | 618 (34.0) | 198 (36.9) |  |
| 2 | 341 (18.7) | 73 (13.6) |  |
| 3 | 89 (4.9) | 23 (4.3) |  |
| 4 | 56 (3.1) | 15 (2.8) | 0.05* |
| Missing | 111 (6.1) | 26 (4.8) |  |
| **Severity by CURB 65 Score:** | | | |
| Low | 821 (45.1) | 312 (58.1) |  |
| Moderate | 559 (30.7) | 124 (23.1) |  |
| Severe | 440 (24.2) | 101 (18.8) | **<0.0001*** |
| **Co-Morbidity:** | | | |
| Malignancy | 171 (9.4) | 49 (9.1) | 0.54 |
| Liver Disease | 36 (2.0) | 14 (2.6) | 0.37 |
| CCF | 128 (7.0) | 17 (3.2) | **0.001** |
| CVA | 159 (8.7) | 20 (3.7) | **<0.0001** |
| Renal disease | 185 (10.2) | 39 (7.3) | **0.04** |
| Diabetes | 307 (16.9) | 69 (12.9) | **0.02** |
| IHD | 210 (11.5) | 41 (7.6) | **0.01** |
| Cognitive impairment | 72 (4.0) | 9 (1.7) | **0.01** |
| Asthma | 187 (10.3) | 60 (11.2) | 0.55 |
| COPD | 467 (25.7) | 95 (17.7) | **<0.0001** |
| Chronic heart disease | 309 (17.0) | 54 (10.6) | **<0.0001** |
| Chronic lung disease | 526 (28.9) | 112 (20.9) | **<0.0001** |
| Hypertension | 457 (25.1) | 114 (21.2) | 0.06 |
| Alcohol | 40 (2.2) | 20 (3.7) | 0.05 |
| Immunosuppression | 80 (4.4) | 28 (5.2) | 0.42 |

**S4 Table:** Characteristics of patients in whom the vaccine status was confirmed via primary care compared to those with self-reports only. *p-value for trend derived from chi-squared test for trend
