# Supplementary material for: Effectiveness of the 23-valent pneumococcal polysaccharide vaccine against vaccine serotype pneumococcal pneumonia in adults: A case-control test-negative design study
Source: PLoS Med. 2020 Oct 23;17(10):e1003326. doi: 10.1371/journal.pmed.1003326 (PMC7584218; doi:10.1371/journal.pmed.1003326)
Supplement: S5 Table — Unadjusted and adjusted results of the primary analysis and the secondary case group analysis in cases against controls. The baseline group for all analysis is the respective control group. Vaccine exposure confirmed and self-reported yes at any point prior to their index admission. Adjusted for sex only. (DOCX) [file pmed.1003326.s007.docx]

### S5 Table: Sub-analysis of healthy 60-75 year olds

|  | **Cases N (%)** | **Controls N (%)** | **Unadjusted Vaccine Effectiveness % (95% CI)** | **Adjusted Vaccine Effectiveness % (95% CI)** | **p-value adjusted analysis** |
| --- | --- | --- | --- | --- | --- |
| **Primary case group – all PPV23:** | | | | | |
| **Number** | 65 | 109 |  |  |  |
| **Not vaccinated** | 40 (61.5) | 63 (57.8) |  |  |  |
| **Vaccinated** | 25 (38.5) | 46 (42.2) | 14 (-61 to 54) | 12 (-67 to 53) | 0.7 |
| **Secondary case group – PPV23/non-PCV13** | | | | | |
| **Number** | 33 | 109 |  |  |  |
| **Not vaccinated** | 23 (69.7) | 63 (57.8) |  |  |  |
| Vaccinated | 10 (35.0) | 46 (42.2) | 40 (-38 to 74) | 39 (-43 to 74) | 0.26 |

**S5 Table:** Sub-analysis of the cohort restricted to 60-75 year olds with no known risk factors.

Unadjusted and adjusted results of the primary analysis andthe secondary case group analysis in cases against controls. The baseline group for all analysis is the respective control group. Vaccine exposure confirmed and self-reported yes at any point prior to their index admission. Adjusted for gender only.
