# Supplementary material for: Effectiveness of the 23-valent pneumococcal polysaccharide vaccine against vaccine serotype pneumococcal pneumonia in adults: A case-control test-negative design study
Source: PLoS Med. 2020 Oct 23;17(10):e1003326. doi: 10.1371/journal.pmed.1003326 (PMC7584218; doi:10.1371/journal.pmed.1003326)
Supplement: S6 Table — Adjusted for age, sex, receipt of seasonal flu vaccination, and presence or absence of the following risk factors: malignancy, cardiac failure, cerebrovascular disease, chronic renal disease, chronic liver disease, diabetes, ischaemic heart disease, COPD, other chronic cardiac disease, other chronic lung disease, hypertension, alcohol dependence, and immunosuppression. ^Adjusted for age, sex, receipt of seasonal flu vaccination. COPD, chronic obstructive pulmonary disease; PPV23, 23-valent pneumococcal polysaccharide vaccine. (DOCX) [file pmed.1003326.s008.docx]

### S6 Table: Sensitivity analysis excluding Serotype 5 from primary case group (All PPV23)

|  | **Cases N (%)** | **Controls N (%)** | **Unadjusted Vaccine Effectiveness % (95% CI)** | **Adjusted Vaccine Effectiveness % (95% CI)** | **p-value adjusted analysis** |
| --- | --- | --- | --- | --- | --- |
| **Number** | 676 | 1640 |  |  |  |
| **Not vaccinated** | 364 (53.9) | 746 (45.5) |  |  |  |
| **Vaccinated** | 312 (46.2) | 894 (54.5) | **28 (14 to 40)** | **26 (7 to 42)*** | **0.01** |

**S6 Table: Sub-analysis excluding serotype 5 from the primary analysis group (all PPV23 serotypes).** Adjusted for age, gender, receipt of seasonal flu vaccination and presence or absence of the following risk factors: malignancy, cardiac failure, cerebrovascular disease, chronic renal disease, chronic liver disease, diabetes, ischaemic heart disease, COPD, other chronic cardiac disease, other chronic lung disease, hypertension, alcohol dependence and immunosuppression. ^Adjusted for age, gender, receipt of seasonal flu vaccination
